# Tyrosine kinase inhibitor AG1024 exerts antileukaemic effects on STI571-resistant Bcr-Abl expressing cells and decreases AKT phosphorylation

**DOI:** 10.1038/sj.bjc.6602190

**Published:** 2004-10-19

**Authors:** E Deutsch, L Maggiorella, B Wen, M L Bonnet, K Khanfir, V Frascogna, A G Turhan, J Bourhis

**Affiliations:** 1 Laboratoire UPRES EA No 27-10, Radiosensibilité des tumeurs et tissus sains, Institut Gustave-Roussy, 39 rue Camille Desmoulins, 94805 Villejuif Cédex, France; 2 INSERM U362, Institut Gustave-Roussy, 39 rue Camille Desmoulins, 94805 Villejuif Cédex, France; 3 Translational Research-Cell Therapy Laboratory, Institut Gustave-Roussy, 39 rue Camille Desmoulins, 94805 Villejuif Cédex, France

**Keywords:** Bcr-Abl, Tyrphostin AG1024, AKT, IGF1, tyrosine kinase, DNA-PKcs

## Abstract

Chronic myelogenous leukaemia (CML) is a clonal malignancy of the pluripotent haematopoietic stem cell, characterised by an uncontrolled proliferation and expansion of myeloid progenitors expressing a fusion oncogene, BCR-ABL, the molecular counterpart of the Ph1 chromosome. The tyrosine kinase (TK) activity of BCR-ABL is known to activate several major signalling pathways in malignant cells, including Ras, JAK/STAT and PI3K/Akt with evidence of proteasome-mediated degradation of other targets such as the DNA repair protein DNA-PKcs and cyclin-dependent kinases inhibitor p27. Targeting these abnormalities by blocking TK of BCR-ABL with STI571 provided a promising approach for the therapy of CML. The recent development of resistance to STI571 illustrates, however, that the use of other TK inhibitors could be of major interest for therapeutic purposes. To this end, the TK inhibitor Tyrphostin AG1024 was used to evaluate effect on regulation of BCR-ABL expression, inhibition of cell proliferation and tumour formation *in vivo* in human and murine BCR-ABL expressing cell lines. Tyrphostin AG1024 was shown to downregulate expression of BCR-ABL and P-Akt, and to upregulate DNA-PKcs expression. In addition, Tyrphostin AG1024 was able to inhibit cell proliferation, and delay tumour growth *in vivo*. Thus, AG1024 is able to interfere with three major targets of BCR-ABL in leukaemic cells. Interestingly, Tyrphostin AG1024 was also effective against cells resistant to STI571 by distinct mechanisms including Bcr-Abl mutation. Therefore, these data suggest that Tyrphostin AG1024 could represent the basis of a novel therapy for STI571 refractory CML.

Chronic myelogenous leukaemia (CML) is a myeloproliferative disorder associated with the presence of the pathognomonic Philadelphia chromosome (Ph), which is found in approximately 95% of patients. CML is a myeloproliferative disorder and it was first described (Nowell and Hungerford, 1960) as a shortened chromosome 22 (within the Bcr locus) and a reciprocal translocation that transfers the c-abl proto-oncogene from the chromosome 9–22 (Lugo *et al*, 1990; McWhirter and Wang, 1991). According to the break within the Bcr locus (Quackenbush *et al*, 2000), three oncogenic BCR-Abl fusion proteins: p190, p210 and P230 can be formed and they all were found to be sufficient to generate leukaemia in humans and murine models (Daley *et al*, 1990; Huettner *et al*, 2000). Indeed, the Bcr-Abl oncogene was found to induce a constitutive increase in the tyrosine kinase (TK) activity (Konopka and Witte, 1985), which is sufficient for its transforming capacity. Recent advances in CML research have highlighted the role of the Bcr-Abl oncoprotein as a molecular abnormality that leads to phenotypic changes in Bcr-Abl expressing cells, and contributes to malignant transformation of cells. In fibroblasts and haematopoietic cells transformation models, it was shown that Bcr-Abl induces mitogenesis (Raitano *et al*, 1997) and growth factor independence (Mandanas *et al*, 1992; Cambier *et al*, 1998; Issaad *et al*, 2000). Studies have demonstrated that the expression of Bcr-Abl fusion proteins protects cells from apoptosis induced by stimuli such as cytokine withdrawal, DNA damage or Fas activation (Bedi *et al*, 1994, 1995; Benito *et al*, 1996). By using Bcr-Abl mutant constructs, separated signalling pathways were found to be involved in the protection of cells from apoptosis and in cell transformation (Cortez *et al*, 1995). It was reported that Bcr-Abl activates several signalling pathways including Ras, Myc, Stat5 and PI3K/Akt (14–20). The antiapoptotic potential of Bcr-Abl was attributed to the increase of Bcl2/BclXL induced by the activation of Stat5 and PI3K/Akt pathways (Sanchez Garcia and Grutz, 1995; Gesbert *et al*, 2000). Furthermore, we previously demonstrated that DNA-PKcs (Deutsch *et al*, 2001) and BRCA1 (Deutsch *et al*, 2003), two major DNA repair proteins, are downregulated by Bcr-Abl in leukaemic cells, these downregulation processes were associated with a major DNA repair deficiency. Thus, we hypothesised that strong resistance to apoptosis along with the DNA repair deficiency induced by Bcr-Abl allow the accumulation of secondary genetic abnormalities leading to the clonal evolution toward blast crisis.

BCR-ABL induces mitogenesis in fibroblasts and haematopoietic cells (Raitano *et al*, 1997). BCR-ABL expression induces growth factor independence in factor-dependent cell lines in a dose-dependent manner (Issaad *et al*, 2000). During the recent years, progresses obtained in the understanding of the BCR-ABL-induced signalling have made possible to correlate signalling abnormalities to phenotypic changes observed in BCR-ABL-expressing cells. Previous studies using BCR-ABL mutant constructs suggested that signals responsible for protection from apoptosis may be separated from those responsible for transformation (Cortez *et al*, 1995). Among the major signalling intermediates activated, there is a clear role of RAS, MYC, STAT5, phosphatidylinositol 3-kinase/Akt pathways for the transforming effects of Bcr-Abl (Cleveland *et al*, 1989; Okuda *et al*, 1994; Skorski *et al*, 1995; Ilaria and Van Etten, 1996). The use of dominant-negative constructs allowed to determine STAT5 and PI-3K pathways to be major signalling pathways responsible of the antiapoptotic potential of BCR-ABL (Gesbert *et al*, 2000), by activation of Bcl2/BclXL (Sanchez Garcia and Grutz, 1995). The central role of Bcr-Abl oncogene in CML suggested Bcr-Abl to be an attractive target for leukaemia therapy. This led to the successful development of new targeted agents such as STI571, which inhibits the TK activity of constitutive activated forms of Abl protein (v-Abl, p210Bcr-Abl, p185Bcr-Abl, Tel-Abl fusion proteins) (Buchdunger *et al*, 1996; Druker *et al*, 1996; Carroll *et al*, 1997). Preclinical and clinical studies have shown that Abl TK inhibition by STI571 was efficient in both p210 Bcr-Abl-positive and p185Bcr-Abl-positive cell lines as well as in primary leukaemia cells obtained from patients having Ph-positive CML and ALL (Beran *et al*, 1998). Further, STI571 was found to inhibit stem cell factor (SCF)-mediated cellular events such as c-Kit autophosphorylation and SCF-mediated activation of MAP kinase and Akt (Buchdunger *et al*, 2000; Heinrich *et al*, 2000). Despite the major antileukaemic effect of STI571 in chronic phase CML, clinical resistance to STI571 treatment was observed in patients with advanced phase diseases and was attributed to the mutations in the ATP-binding site of the Bcr-Abl protein, which alters drug binding and thus its inhibitory effects (La Rosee *et al*, 2002; Roche-Lestienne *et al*, 2002). Therefore, antileukaemic strategies targeting TK inhibition by new agents alone or in combination with STI571 need to be investigated. Bcr-Abl has been shown to be leukaemogenic in a variety of animal models (Daley *et al*, 1990) essentially by its deregulated TK activity (Lugo *et al*, 1990). PI3K and Akt have been linked to enhanced cell survival through the phosphorylation and subsequent inhibition of the proapoptotic molecule Bad (Neshat *et al*, 2000). Bcr-Abl regulates the expression of p27Kip1 in a proteasome-dependent manner and through activation of PI3K and Akt (Gesbert *et al*, 2000). Tyrphostin AG1024 has been reported to induce apoptosis and to enhance radio sensitivity by downregulating PI3K/Akt signalling pathway (Wen *et al*, 2001). Tyrphostin AG1024 is a TK inhibitor which inhibits phosphorylation of IGF1R and MEK and this compound is also able to induce pRb degradation (Baserga, 2000). In this study, we investigated in Bcr-Abl expressing cells the effect of this inhibitor on Akt phosphorylation and its consequences on Bcr-Abl and the DNA repair protein DNA-PKcs. Furthermore, we investigated whether Tyrphostin AG1024 induced an antileukaemic effect both *in vitro* and in nude mice as well as the effectiveness of Tyrphostin AG1024 against STI571 resistant cells.

## MATERIALS AND METHODS

### Cell culture

UT7 is a pluripotent human erythroleukaemia cell line whose growth is dependent on GM-CSF (Ahmed *et al*, 2000). BCR-ABL-expressing counterparts of this cell line have been obtained by the use of enforced expression of BCR-ABL by either retroviral infection or transfection as previously described (Ahmed *et al*, 2000). UT7-9 cells stably express high levels of the p210 fusion protein and are GM-CSF-independent for their growth. Murine Ba/F3 cell line and its BCR-ABL-expressing counterparts have also been previously described. Human UT7, UT7-9 and murine Ba/F3 and Ba/F3-p210 cells were grown in RPMI 1640 with 10% FCS. A measure of 10 ng ml^−1^ recombinant human granulocyte-macrophage colony-stimulated factor (rhGM-CSF) (R&D Systems, Minneapolis, MN, USA) was added to the culture media of UT7 cells and 10% WEHI conditioned medium as a source of interleukin 3 (IL-3) was added to the culture medium of Ba/F3 cells.

### Imatinib mesylate (Gleevec) resistant cells

#### Cell lines

CML blast-crisis K562 and the partially STI571 resistant K562R cells were kindly provided by Dr Weisberg, Dana Farber Cancer Institute, Boston, USA and cultured in DMEM medium supplemented with 10% FCS. These cell lines exhibit Bcr-Abl overexpression.

#### Patients derived cells

Bone marrow and/or peripheral blood samples from two patients were obtained after informed consent as a protocol study sanctioned by the local institutional review board (IRB). Cells were derived from a splenectomy sample of a Bcr-Abl-positive CML-blast crisis patient, who relapsed receiving imatinib mesylate (Gleevec). Mononuclear cells were isolated and used to purify CD34+ leukaemia blasts, as previously described (Deutsch *et al*, 2001).

### Assessment of apoptosis after treatment with AG1024

After exposure to Tyrphostin AG1024, apoptotic changes were detected using fluorescein isothiocyanate (FITC)-annexin V, which binds to phosphatidylserine exposed on the outer leaflet of apoptotic cell membranes. Propidium iodide (PI) staining was used for the discrimination between apoptotic and necrotic cells among the annexin V1 cells. Cells were washed and resuspended in 490 ml binding buffer solution (Annexin V-FITC Kit, Immunotech, Fullerton, CA, USA). Annexin V-FITC (5 ml) and 5 ml PI were then added to the cell suspension for 10 min followed by FACS analysis.

### Western blot

Cells were washed with cold PBS, and lysed in lysis buffer (50 mM HEPES pH 7.4, 15 mM NaCl, 0.1% Tween-20, 10% Glycerol, 2.5 mM EGTA, 1 mM EDTA, 1 mM phenylmethysulphonyl fluoride, and inhibitors for proteases). Protein concentration was determined by using Bradford assay. Equal amounts of proteins were loaded into wells and separated by 5–12% SDS–PAGE gel. The proteins were transferred onto nitrocellulose membranes, blocked overnight at 4°C in TBS-T solution, containing 5% nonfat milk, and incubated with primary monoclonal antibodies: ABL (Oncogene, Cambridge, MA, USA), DNA-PKcs (Neomarker, Fremont, CA, USA), phospho-Akt (New England Biolab, Beverly, MA, USA), Akt (New England Biolab), BRCA1 (Santa Cruz Biotechnology, Santa Cruz, CA, USA). After washing, the appropriate secondary antibody conjugated to horseradish peroxydase (Jackson ImmunoResearch Lab, West Grove, PA, USA) was probed. Membranes were developed using the enhanced chemiluminescence detection system (Amersham Biotech Company, Piscataway, NJ, USA). *β*-Actin (Sigma) was used to control protein loading. Densitometry analysis of the relative intensity of spots has been performed in order to quantify the expression of Bcr-Abl after treatment of both K562 and K562R cells with Tyrphostin AG1024 using Mac Bas2 software.

### Cell proliferation assays

To study the inhibitory effect of Tyrphostin AG1024 on cell growth, 10^6^ cells were plated and the different drug concentrations (2, 5, 10 and 50 nM) were added to the medium. At the indicated time, cell number and viability were estimated by trypan blue assay.

### Clonogenic assays

Both UT7 clones, K562 cells and cells derived from patients were transplanted in methylcellulose (Stem Cell Technologies) in the presence of 10 ng ml^−1^ rhGM-SCF for UT7 cells or 10% WEHI for Ba/F3 cells and graded concentration of Tyrphostin AG1024. The surviving fraction was determined by measuring the viability of colony-forming unit compared with the corresponding untreated controls, data represent the mean of three independent experiments performed in triplicate. Colonies were estimated 10–14 days after the treatments.

### *In vivo* experiments

Female nude mice (6–8 weeks old) were purchased from Janvier CERT 53940 Le Genest St Isle, France. Animals used in this study were maintained in facilities in accordance with current regulations and observing ‘Principles and Guidelines for the Use of Animals in Research’ Issued by the French government according to the European community rules. 10^6^ Ba/F3-p210 cells in 0.1 ml of were implanted subcutaneously into the right flank of mice and the animals were randomly assigned to control or treatment group. Mice were injected i.p. with Tyrphostin AG1024 (30 *μ*g in 100 *μ*l) once per day for 2 weeks in the treatment group and with 100 *μ*l PBS in the control. The mice were inspected daily for the tumour growth and any other signs of disease or distress. The tumour volume was calculated from the greatest transverse (width) and longitudinal (length) diameter of the tumour using the formula: tumour volume=length × width^2^/2.

### Statistical analyses

The data are presented as mean values±s.e. The surface areas of the primary tumours and the body weights were compared by paired student's *t*-test.

## RESULTS

### Downregulation of BCR-ABL expression by Tyrphostin AG1024

**Figure 1:**
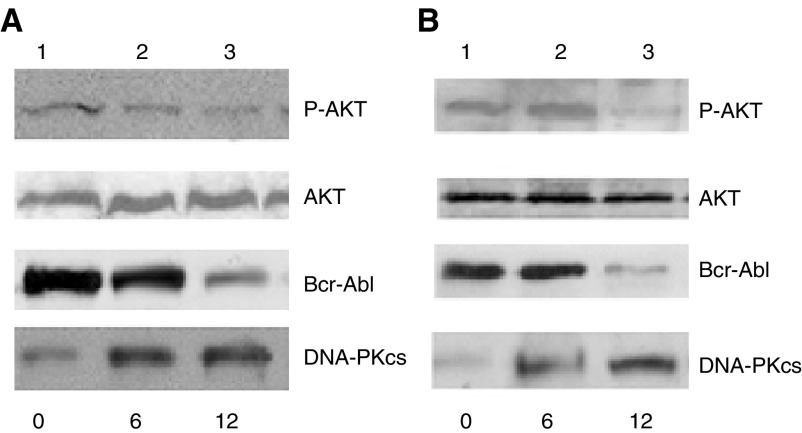
Tyrphostin AG1024 downregulates phospho-Akt, Bcr-Abl and upregulates DNA-PKcs. (**A**) UT7-9 and (**B**) Ba/F3-p210 cells were treated with Tyrphostin AG1024 2 *μ*M, harvested at a definite time interval. Lane 1: control; lane 2: 6 h; lane 3: 12 h.

Immunoblot analysis was used to assess the expression of the fusion protein Bcr-abl in UT7-9 and Ba/F3-p210 cells after treatment with Tyrphostin AG1024. As shown in Figure 1

, expression of Bcr-Abl was downregulated by Tyrphostin AG1024 in UT7-9 and Ba/F3-p210 cells.

### Tyrphostin AG1024 decreases the expression of Phospho-Akt (ser473) in BCR-ABL-expressing cells

Further experiments were designed to measure the expression of phosphorylated forms of AKT. As shown in Figure 1, phospho-Akt (ser473) levels were downregulated by Tyrphostin AG1024, in a time-responsive manner but the amount of Akt remained the same in UT7-9 and Ba/F3-p210 cells. The expression of phospho-Akt was not influenced by the exposure to Tyrphostin AG1024 in their parental cells UT7 and Ba/F3-P (data not shown).

### Restoration of the DNA-PKcs levels in BCR-ABL-expressing cells by Tyrphostin AG1024

PI3K and Akt have previously reported to play essential roles in Bcr-Abl transformation, and our previous work showed that DNA-PKcs is downregulated by Bcr-Abl (23). Experiments were designed to assess whether the expression of DNA-PKcs was influenced by the TK inhibitor Tyrphostin AG1024. As shown in Figure 1, treatment with Tyrphostin AG1024 induced an upregulation of DNA-PKcs levels in UT7-9 and Ba/F3-p210 cells (Figure 1).

### Decreased clonogenic survival and proliferation of murine Ba/F3 and human UT7 BCR-ABL-expressing cell lines after exposure to Tyrphostin AG1024

**Figure 2:**
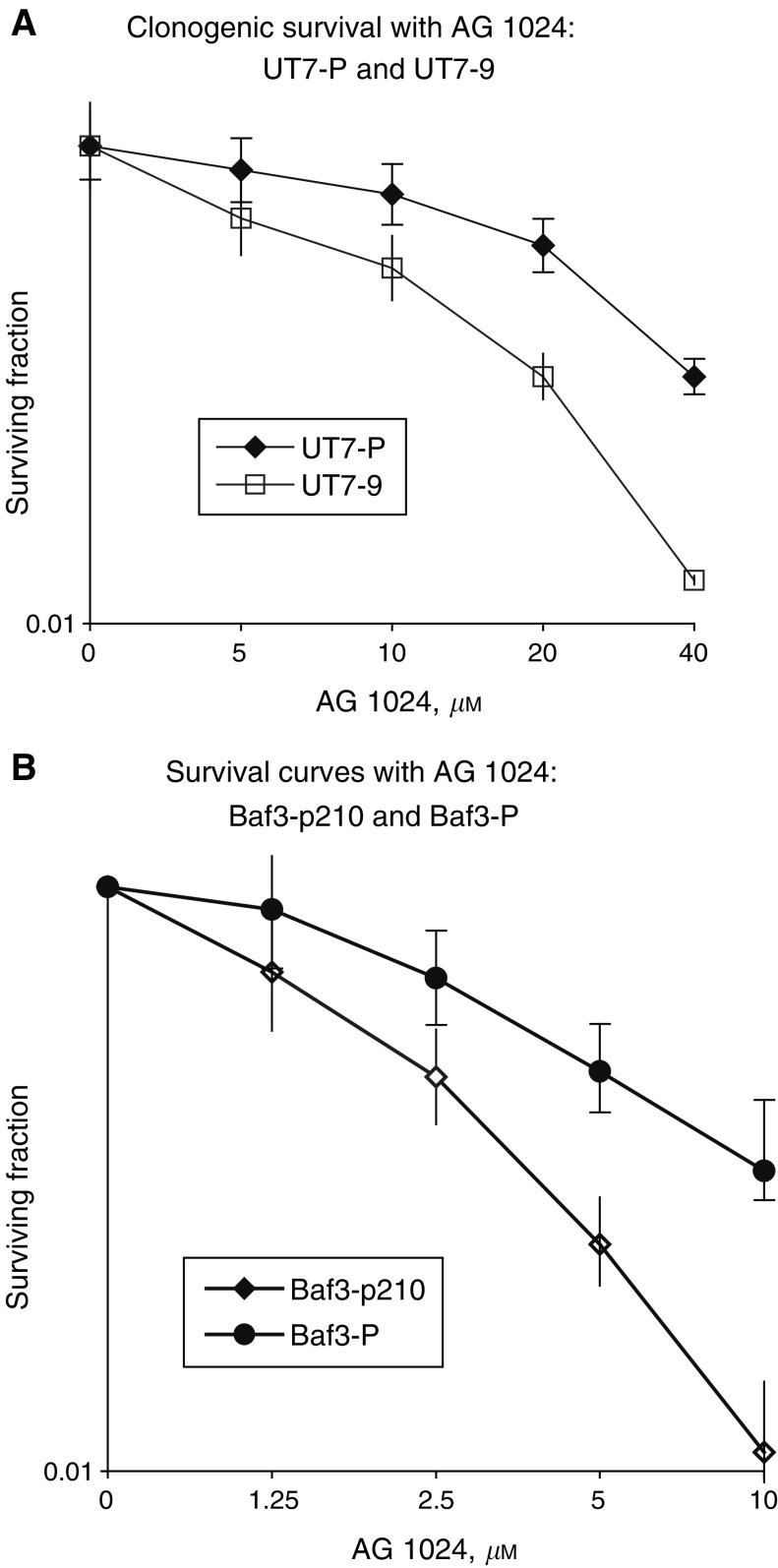
Clonogenic survival after exposure to Tyrphostin AG1024 of UT7 and UT7-9 cells (**A**), Ba/F3 and Ba/F3-p210 (**B**), respectively. Experiments were repeated three times, each experiment was triplicated. Error bars represent the 95% confidence interval.

**Figure 3:**
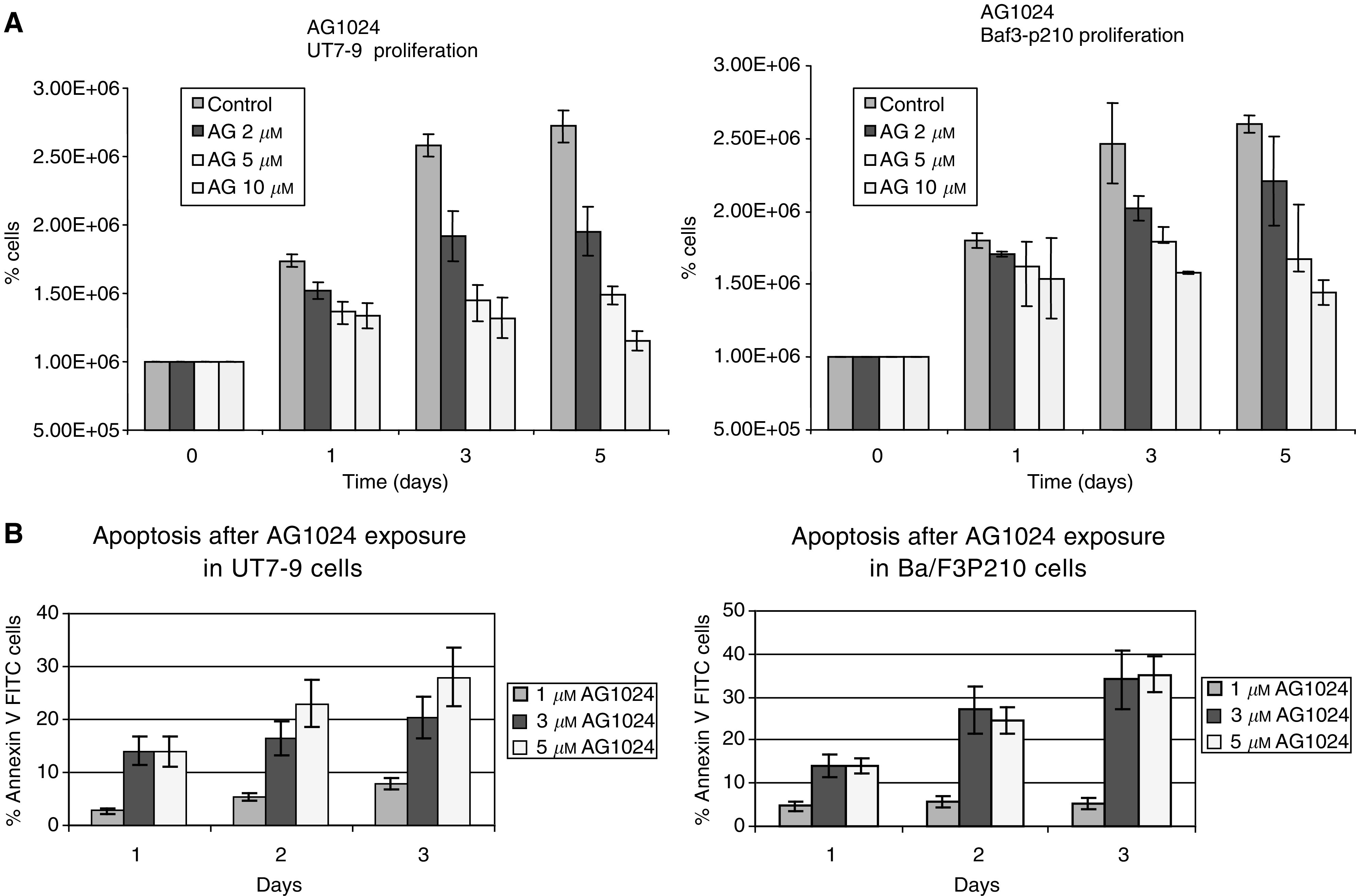
Antiproliferative effect of Tyrphostin AG1024 in UT7-9 and Baf3-p210 cells. (**A**) Time course of cell proliferation in different concentrations of Tyrphostin AG1024 in UT7-9 and Baf3-p210 cells, each experiment was carried out triplicate and repeated three times. Error bar shows the mean±s.e. (**B**) A time course of apoptosis after exposure to different concentrations of Tyrphostin AG1024 in UT7-9 and Baf3-p210 cells, each experiment was carried out in triplicate and repeated three times. Error bar shows the mean±s.e.

Clonogenic survival assay showed that Bcr-Abl expressing cells exhibited higher colony formation inhibition than parental cells (Figure 2A and B

). The effect of Tyrphostin AG1024 on cell proliferation was evaluated in UT7-9 and Ba/F3-p210 cells. Proliferation curves showed a dose-dependent inhibition of cell proliferation after treatment with Tyrphostin AG1024 (in Figure 3A

). The effects of Tyrphostin on cell proliferation and clonogenic survival was associated with the induction of apoptosis (Figure 3B).

### *In vivo* inhibition of Bcr-Abl expressing cells growth with AG1024

**Figure 4:**
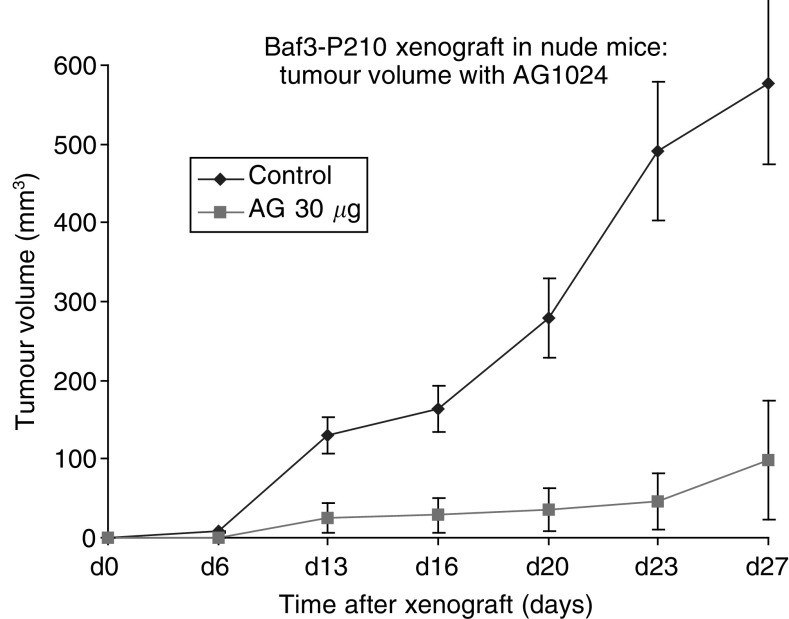
Antitumour effect of Tyrphostin AG1024 Ba/F3-p210 cells. After injection with 10^6^ cells/0.1 ml nude mice were injected after 6 days with PBS 0.1 ml (control) or Tyrphostin AG1024 30 *μ*g/0.1 ml (0.1 ml of a 980 *μ*M solution) in treatment group i.p. per day for 10 days. The experiment was repeated two times with five animals in each group. Error bars represent the mean±s.e. of the ratio.

In order to complete, our results showing antiproliferation potential of Tyrphostin AG1024 in Bcr-Abl expressing cells *in vitro*. We investigated the antitumour effect of Tyrphostin AG1024 *in vivo* in nude mice on Ba/F3-p210 xenografts. The tumour growth was significantly delayed when mice were treated with Tyrphostin AG1024 (Figure 4

).

### Efficacy on Tyrphostin AG1024 on Bcr-Abl expressing STI571 resistant cells

**Figure 5:**
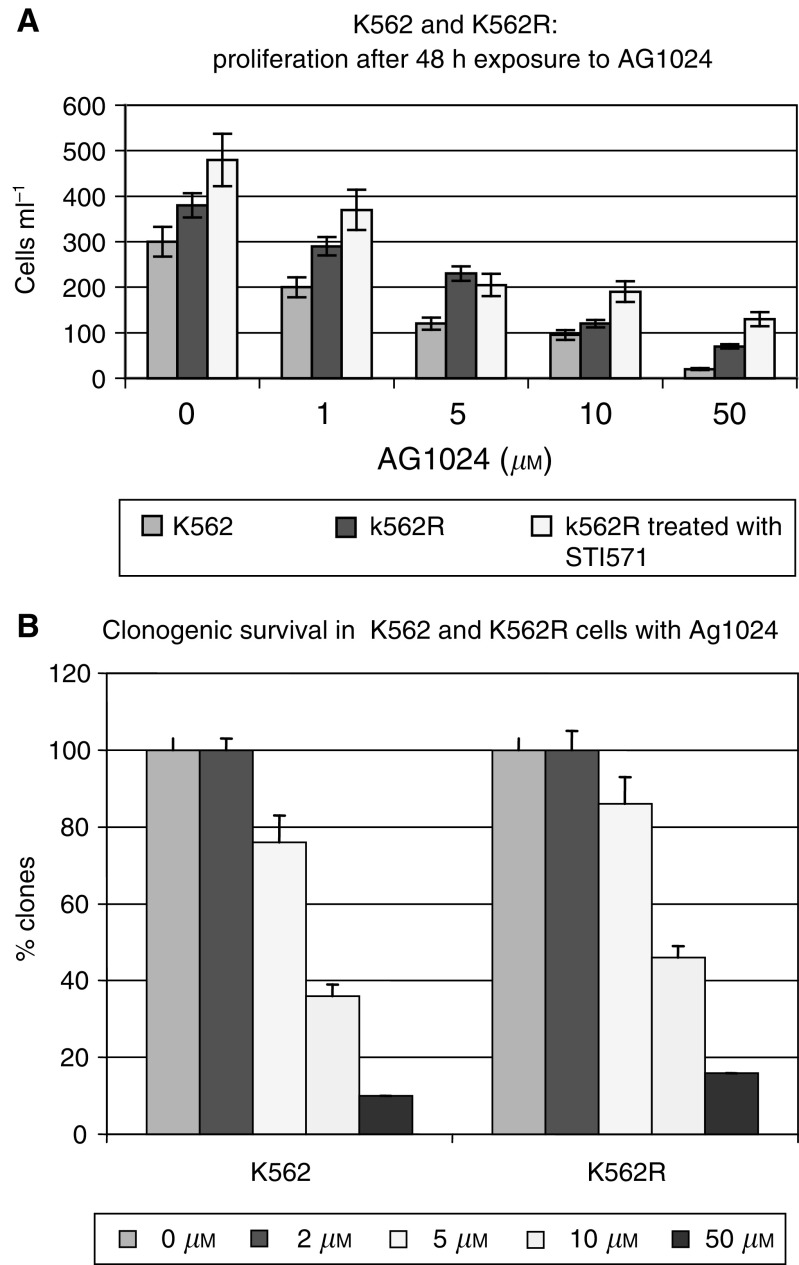
Effects of AG1024 exposure in the parental K562 and in the STI571 resistant K562R cell line. (**A**) Effects on cell proliferation after 48 h exposure to AG1024 on K562 and K562R cell lines. Experiments were repeated three times, each experiment was in triplicate. Error bars represent the 95% confidence interval. (**B**) Effects of AG1024 on K562 and K562R colony formation after 10 days culture in a semisolid. Experiments were repeated three times, each experiment was in triplicate. Error bars represent the 95% confidence interval.

**Figure 6:**
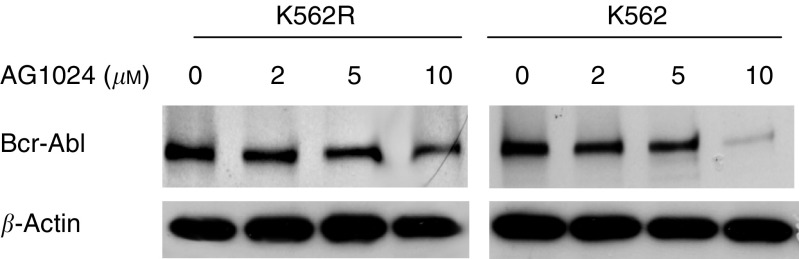
Western blot analysis of Bcr-Abl expression in K562 and K562R cells after exposure to AG1024. *β*-Actin was used as a loading control.

Proliferation curves and colony formation assays for K562 (STI571 sensitive) and K562R (STI571 resistant) cell lines were used to evaluate the effects of Tyrphostin AG1024 on cell proliferation and clonogenic survival in Bcr-Abl expressing cells resistant to STI571(Figure 5

). Exposure to AG1024 caused a marked inhibition of proliferation (Figure 5) and colony formation, this effect was observed independently of the sensitivity of K562 and K562R cells to STI571. Interestingly, this effect correlates with a decrease in Bcr-Abl protein expression in a dose-dependent manner (Figure 6

). Densitometry analysis showed a reduction in Bcr-Abl expression of, respectively, 38, 43 and 61% in K562R and 41, 59 and 78% in cells treated with 2, 5 and 10 *μ*M Tyrphostin AG1024.

### CML cells from STI571 resistant patients are sensitive to Tyrphostin AG1024

**Figure 7:**
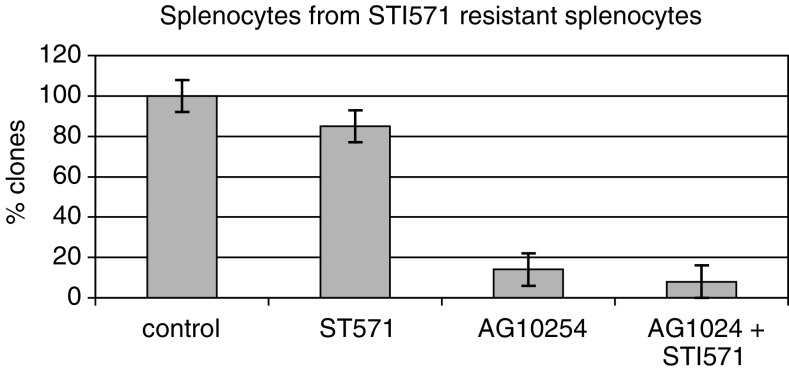
Effects of AG1024 on colony formation after 10 days culture in a semisolid methylcellulose medium on splenocytes obtained from a CML-BC patients refractory to STI571. Cells were treated with STI571 at 1 *μ*M, AG1024 at 50 *μ*M. Error bars represent the mean±s.e. of the ratio.

**Figure 8:**
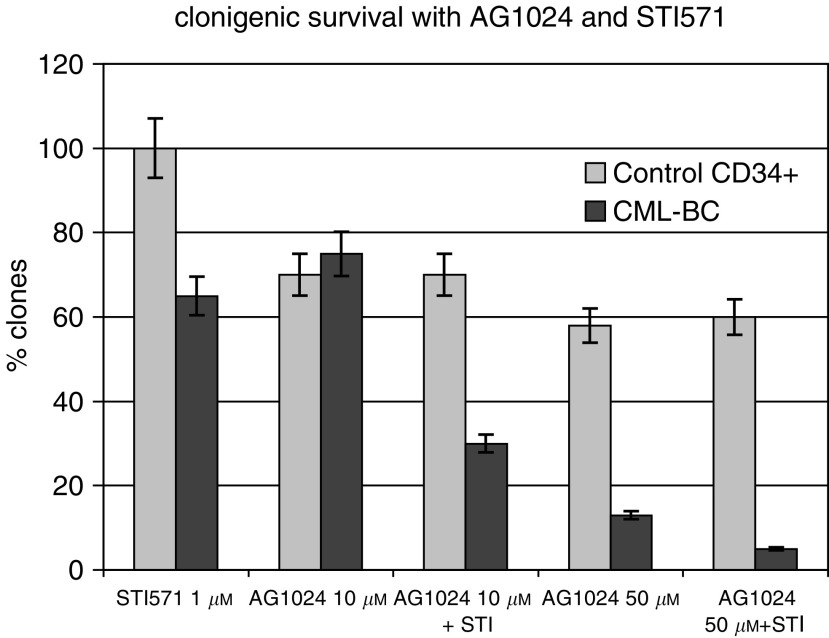
Effects of AG1024 on colony formation after 10 days culture in a semisolid methylcellulose medium on cells obtained from two CML-BC patients refractory to STI571 and from control CD34+ cytapheresis cells. The percentage of clones is obtained by dividing the percentage of clones when cells are treated divided by the percentage of clones when cells are untreated. The diagram shows mean values with the two patients, each experiment was carried out in triplicate. Error bars represent the mean±s.e. of the ratio.

Clonogenic survival with Tyrphostin AG1024 and STI571 was studied in order to evaluate the effects of this combination on clonogenic CML cells obtained from patients relapsing after STI571 treatment. The AG1024 and STI571 were added directly to the methylcellulose cultures at the time of clonogenic assay and the results were compared to untreated controls. We observed a significant inhibition of clonogenic activity with Tyrphostin AG1024 in two different cases, primary leukaemic cells from two CML from patients clinically refractory to STI571 treatment (Figure 7

) and in splenocytes derived from one CML patient refractory to STI571 (Figure 7 and Figure 8

respectively). Interestingly, AG1024 concentration of 10 and 50 *μ*M are more effective on CML cells than on normal CD34 cells, suggesting the existence of a differential effect of Tyrphostin AG1024 (Figure 7).

## DISCUSSION

Our results have shown that the proliferation of Bcr-Abl expressing cells was inhibited after Tyrphostin AG1024 treatment both in human and murine cell lines *in vitro*. This growth inhibitory effect was also effective *in vivo*, in the murine Ba/F3 Bcr-Abl-p210 leukaemia xenograft model without inducing significant toxicity. Molecular studies of protein expression showed that exposure to AG1024 was associated with a decrease in the phosphorylated form of Akt (Ser 473), which is the main downstream component of the PI3K/Akt pathway responsible of apoptosis inhibition and cell proliferation. For the first time to our knowledge we show that the TK inhibitor, Tyrphostin AG1024, increases the levels of DNA-PKcs levels with a concomitant decrease in the expression of Bcr-Abl, suggesting that the use of AG1024 could be beneficial for the restoration of DNA-PKcs mediated repair in CML cells. This effect was associated with a concomitant decrease in the expression of the Bcr-Abl protein in both human and murine UT7 and Ba/F3 models. One of the major characteristics of Ph1+ cells is the presence of genetic instability, associated with the progression of the disease from the chronic to the acute phase (Deutsch *et al*, 2001). During the last years, intense research was investigated to understand the transformation and malignant progression of leukaemic cells. Recently, a relationship between the abnormalities of DNA repair proteins and the oncoprotein Bcr-Abl was suggested (Deutsch *et al*, 2001). Indeed, it was established that BCR-ABL increases the mutation frequency in haematopoietic cells by inducing a ‘mutator phenotype’. Abnormalities of RAD51 protein expression have been linked to chemo resistance of CML cells (Slupianek *et al*, 2001; Slupianek *et al*, 2002). The TK dependent nature of these DNA repair abnormalities suggest that the ability to restore them by the use of therapeutic agents such as STI571 could therefore have a therapeutic impact, especially if this could lead to the retardation of the occurrence of blast crisis. The inhibitory effect of AG1024 was also observed in STI571 resistant cell lines (K562R). Exposure to AG1024 in these STI571 resistant cells was associated with a decrease in Bcr-Abl protein expression both in K562 and K562R cell lines, but this phenomenon was observed at higher Tyrphostin AG1024 concentrations than in the UT7 and Ba/F3 cell lines. Bcr-Abl expressing cells from CML patients refractory to STI571 treated with Tyrphostin AG1024 also showed a marked decrease in clonogenic survival. Tyrphostin AG1024 is a TK inhibitor that has originally been reported to be specific for the IGF1 receptor, it has recently been shown that Tyrphostin AG1024 could inhibit phosphorylation of other TK such as MEK, that is part of the MAPK signalling pathway. Moreover, exposure to Tyrphostin AG1024 is also associated to both decrease in the phosphorylated form and degradation of pRb (vonWillebrand *et al*, 1998). Recently, Typhostin AG1024 has been reported to induce apoptosis and to enhance radiosensitivity by downregulating PI3K/Akt signal pathway (Wen *et al*, 2001). The relative importance of IGFR signalling during Bcr-Abl tumorigenic process still has to be studied. IGF1R is a membrane TK receptor ubiquitously expressed except on mature B cells and hepatocytes (Valentinis and Baserga, 2001). Insulin-like growth factors (IGFs) have been shown to potently stimulate cell proliferation and to inhibit cell death (Komatsu *et al*, 1997). Several intracellular signalling pathways have been identified that are activated in response to IGF stimulation. One of them is the phosphatidylinositol 3-kinase (PI3K) pathway. In response to IGF stimulation, activated PI3K converts phosphatidylinositol 4,5-bisphosphate to phosphatidylinositol 3,4,5-trisphosphate, which results in subsequent activation of the pleckstrin homology domain-containing serine/threonine kinases PDK1 and Akt. Oncogenes like RAS, SV40 (Sell *et al*, 1993), the E7 viral oncoprotein (Steller *et al*, 1996) as well as TK receptors EGFR, PDGFR (Coppola *et al*, 1994) fail to operate cellular transformation in IGF1R −/− fibroblasts. Whether IGF1R seems to be required for Bcr-Abl transformation has to be studied. In conclusion, Tyrphostin AG1024 was found to downregulate the expression of Bcr-Abl and P-Akt, and to upregulate the DNA-PKcs protein expression in BCR-ABL expressing cells. *In vitro* Tyrphostin AG1024 was found to inhibit cell proliferation and *in vivo* to induce tumour growth delay. In addition, an antiproliferate effect of Tyrphostin AG1024 was observed in haematopoietic cells resistant to STI571 and in primary CML cells derived from patients resistant to STI571. This effect of Tyrphostin AG1024 was observed independently of the mechanism of resistance to STI571, by Bcr-Abl overexpression or Bcr-Abl gene ATP pocket mutation. Therefore, this study might provide a rationale basis for a new strategy to circumvent STI571 resistance by the combined use of two distinct TK inhibitors in a curative or a preventive setting.
